# Cisplatin and carboplatin pharmacokinetics in a pediatric patient with hepatoblastoma receiving peritoneal dialysis

**DOI:** 10.1007/s00280-020-04130-z

**Published:** 2020-08-20

**Authors:** A. Laura Nijstad, Natasha K. A. van Eijkelenburg, Kathelijne C. J. M. Kraal, Marieke J. M. Meijs, Clara T. M. M. de Kanter, Marc R. Lilien, Alwin D. R. Huitema

**Affiliations:** 1 Department of Clinical Pharmacy, Division of Laboratory Medicine and Pharmacy, University Medical Center Utrecht, Utrecht University, Utrecht, The Netherlands; 2 Princess Máxima Center for Pediatric Oncology, Utrecht, The Netherlands; 3 Department of Pharmacy and Pharmacology, Netherlands Cancer Institute, Amsterdam, The Netherlands; 4 Department of Pediatric Nephrology, Wilhelmina Children’s Hospital, University Medical Center Utrecht, Utrecht, The Netherlands

**Keywords:** Cisplatin, Carboplatin, Hepatoblastoma, Pharmacokinetics, Peritoneal dialysis

## Abstract

**Purpose:**

Cisplatin and carboplatin are frequently used drugs in the treatment of pediatric hepatoblastoma. Dosing guidelines for these drugs in children requiring peritoneal dialysis are lacking. Here, we describe the case of a 3-year-old boy with pre-existing end-stage renal disease on peritoneal dialysis, requiring treatment with cisplatin and carboplatin for hepatoblastoma.

**Methods:**

Pharmacokinetic data were generated to support clinical dosing decisions, with the aim of adequate exposure and minimal toxicity. In the first chemotherapy cycle, 25% of the standard cisplatin dose and 75% of the carboplatin dose, calculated using the pediatric Calvert formula, were administered. Free platinum concentrations were determined in plasma ultrafiltrate and dialysate samples drawn after administration of cis- and carboplatin.

**Results:**

Cisplatin was well tolerated and the observed AUC of cisplatin were 15.3 and 14.3 mg/L h in cycles 1 and 3, respectively. The calculated AUC of carboplatin in cycle 1 (9.8 mg/mL min) exceeded target AUC of 6.5 mg/mL min and toxicity was observed; therefore, the dose was reduced in cycles 2 and 3. The observed AUC in cycles 2 and 3 was 5.4 and 5.7 mg/mL min respectively. Platinum concentrations in the dialysate showed that 3–4% of the total dose of cisplatin and 10–12% of the total dose of carboplatin were excreted via peritoneal dialysis. Chemotherapy enabled extended hemihepatectomy and complete remission was achieved.

**Conclusion:**

This report shows that it is feasible to measure AUCs for both drugs and to individualize the dose of these drugs according to the PK results and clinical parameters. Our advice for future cases would be to calculate the starting dose of carboplatin using the (pediatric) Calvert formula, assuming a dialytic clearance of zero, and to adjust the dose if required, based on therapeutic drug monitoring.

## Introduction

Hepatoblastoma is the most common malignant liver tumor in the pediatric population [1]. In most of the cases, (neo)adjuvant chemotherapeutic treatment of hepatoblastoma in children consists of platinum-based therapy, such as cisplatin and carboplatin. After administration, these drugs bind irreversibly to proteins and tissue. Free platinum is considered the active form in terms of antitumor effect and toxicity. Free platinum is mainly eliminated by the kidneys.

In rare cases, children with hepatoblastoma have concomitant kidney failure or a type of kidney disease. Impairment of renal function diminishes platinum clearance, causes an increase of the toxicity of platinum compounds and thereby complicates the treatment of children with hepatoblastoma. Dosing guidelines recommend a reduction of the dose or even omitting therapy with platinum derivatives in patients with renal impairment to prevent further nephrotoxicity. With the exception of a few case reports [2–4], there is no information available on the dosing of cisplatin and carboplatin in children with end-stage renal disease requiring peritoneal dialysis. If platinum drugs are excluded, treatment options are scarce, so more information about the dosing of these agents in patients on renal replacement therapy is needed.

As recommended by Labaki et al. [5], drugs undergoing significant renal elimination should be administered with caution in peritoneal dialysis patients with close monitoring of adverse events, dose reductions should be applied when using anti-cancer treatments that are typically excreted by the kidneys, and pharmacokinetic studies should be performed when available and dose adjustments applied when necessary, even in the absence of any toxicity.

This report describes the treatment of hepatoblastoma with cisplatin and carboplatin in a pediatric patient with pre-existing end-stage renal disease on nocturnal intermittent peritoneal dialysis (NIPD).

## Patient and methods

### Patient

The patient is a 3-year-old boy with end-stage renal disease due to an atypical hemolytic uremic syndrome (HUS) following an influenza A infection. He remained anuric and peritoneal dialysis was started at the age of 5 months. He presented with an unexplained decline of his hemoglobin level for which an abdominal ultrasound was performed. This revealed a large mass in the left lobe of the liver of approximately 12 × 6 × 13 cm. An abdominal CT confirmed the presence of a large hepatic mass involving segments 2, 3 and 5 of the liver, staging to a PRETEXT III. There were no distant metastases detected. Alpha-fetoprotein (AFP) was elevated to 12,500 µg/L (reference range 0.8–4.5 µg/L). Biopsy of the mass revealed epithelial hepatoblastoma.

### Dialysis prescription

The patient was on a nocturnal intermittent cycler-assisted peritoneal dialysis schedule. The dialysis prescription was not changed for the oncological treatment, but dialysate glucose composition was adapted dependent on fluid status of the patient. The total dialysate volume prescribed was 4200 ml/day (9.24–9.35 L/1.73 m^2^/day). Dialysis time was 12 h/treatment, with seven exchanges per treatment. Daily ultrafiltration varied between 223 and 521 ml/treatment. Dialysis adequacy was monitored during treatment by measurement of *K*·*t*/*V* for urea and creatinine clearance. *K*·*t*/*V* was 2.38/week and creatinine clearance was 36 L/week. The patient had no residual renal function.

### Treatment

He commenced with chemotherapy according to the intermediate group of the 'Pediatric Hepatic International Tumor Trial' (PHITT) SIOPEL 3 high-risk (HR) treatment regimen, as there was some concern about extrahepatic extension and this regimen was deemed less toxic than others.

The proposed treatment schedule included cisplatin, carboplatin and doxorubicin. The cisplatin dose according to protocol was 80 mg/m^2^ on day 1 and the carboplatin dose was 500 mg/m^2^ on day 15 as an intravenous (iv) infusion. In view of the impaired renal clearance and based on the case report on Sebestyen et al. [4], the dose of cisplatin was reduced to 20 mg/m^2^ (25% of the recommended dose) and administrated over 6 h. The carboplatin dose was calculated using the formula of Newell et al. [6]. In adults, the carboplatin dose is calculated using the Calvert formula [7]. Calvert showed that the renal clearance of carboplatin is linearly related to the glomerular filtration rate (GFR) and designed a formula to calculate an individual dose using a target area under the curve (AUC). Newell et al. developed a similar formula which is suitable for children:

$${\text{Carboplatin}}\,{\text{dose}}\,({\text{mg}}) = {\text{target}}\,{\text{AUC}} \times ({\text{GFR}} + (0.36 \times {\text{BW}})),$$

where BW equals the body weight in kg. According to Newell et al., a carboplatin dose of 500 mg/m^2^ equals an AUC of approximately 6.5 mg/ml min. With the formula and an estimated dialysis creatinine clearance of 5 ml/min, a dose of 64 mg was calculated, which was reduced to 75% (48 mg, which equals 75 mg/m^2^). This was done for extra safety, since it was unknown if the estimated dialysis creatinine clearance exactly corresponded to the carboplatin clearance. Carboplatin was administrated intravenously in 1 h. Free plasma concentrations of cisplatin and carboplatin were measured after the platinum cycles to further individualize the dose. In the first cycle, doxorubicin was given in a 100% dose of 30 mg/m^2^ on days 15 and 16. Treatment-related toxicities were graded conforming to Common Terminology Criteria for Adverse Events version 5.0 [8], and grade 3 toxicity or higher was recorded.

### Pharmacokinetic analysis

Blood samples for the determination of platinum pharmacokinetics were collected at three or four time points after infusion of cis- and carboplatin up to 24 h after administration. For this purpose, an ultrafiltrate was prepared from plasma samples. In addition, samples were taken from the dialysate after the first NIPD cycle after administration of platinum. Free and total platinum concentrations were measured in this ultrafiltrate and dialysate using a validated inductively coupled plasma mass spectrometry (ICP-MS) method as described by Brouwers et al. [9]. AUC_0–inf,free_ for each cycle was calculated using the trapezoid method. R (version 3.6.1) was used for data handling and visualization [10].

## Results

### Treatment and dose adjustment

**Fig. 1:**
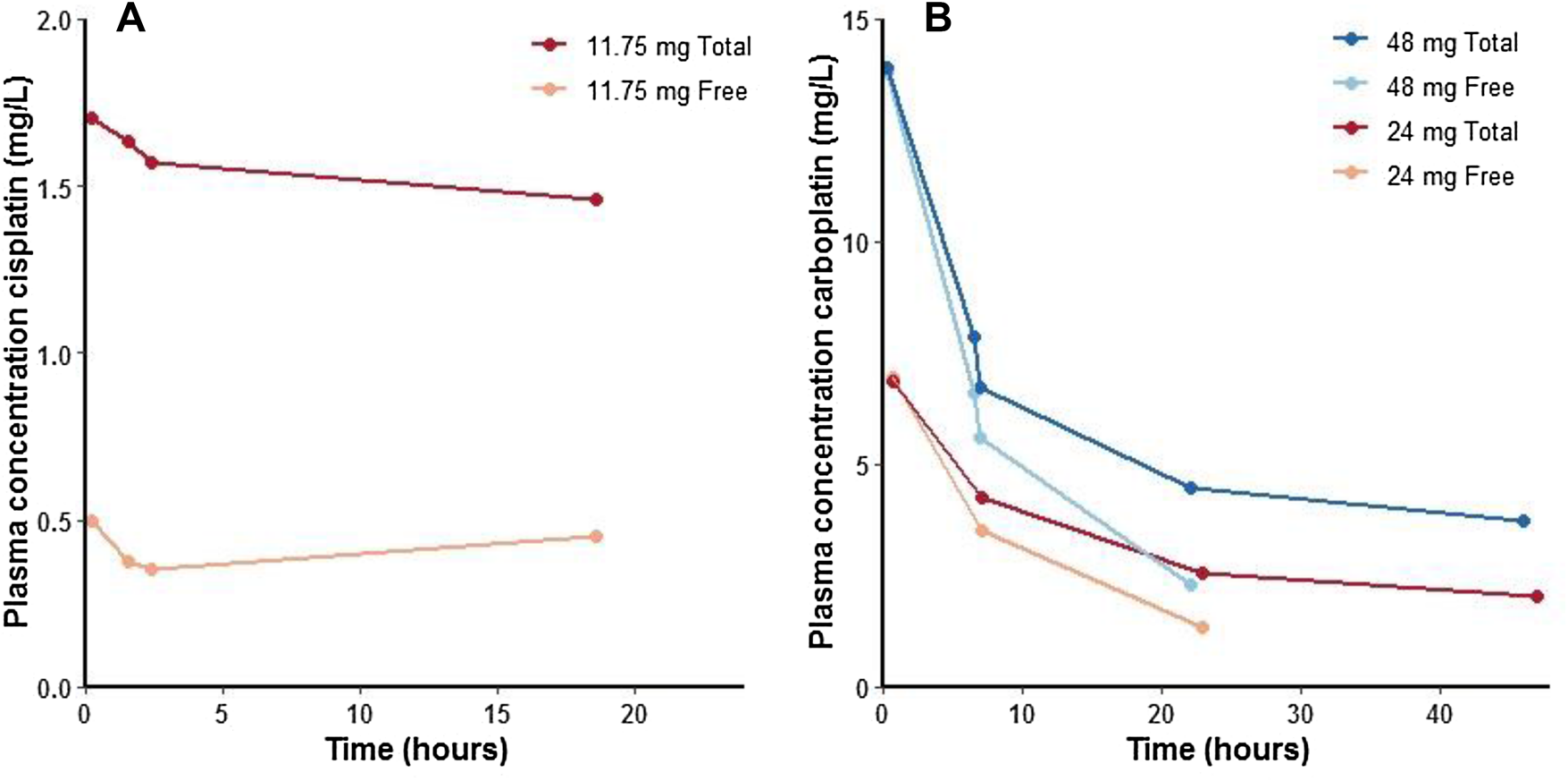
Plasma concentration–time curves for cisplatin (**a**) and carboplatin (**b**)

Blood samples were collected after two cisplatin cycles and three carboplatin cycles. The PK parameters of each cycle are displayed in Table 1. The plasma concentration time curves are displayed in Fig. 1. The AUC_0–inf,free_ of cisplatin following the first dose of 20 mg/m^2^ was 15.3 mg/L h. No significant side effects were noted after this first cycle of cisplatin. The AUC_0–inf,free_ of carboplatin following the first dose of 48 mg (75 mg/m^2^) was found to be 9.8 mg/mL min, which exceeds the target AUC of 6.5 mg/mL min. Subsequently, severe clinical toxicity was observed, consisting of grade 3 mucositis.

**Table 1:** Pharmacokinetic parameters and tolerance for cisplatin and carboplatin

	Total dose (mg)	Dose (mg/m^2^)	AUC_0–inf,free_	Tolerance
*Cisplatin*				
Cycle 1	11.75	20	15.3 mg/L h	No side effects
Cycle 3	11.75	20	14.3 mg/L h	No side effects
*Carboplatin*				
Cycle 1	48	75	9.8 mg/mL min	Grade 3 mucositis
Cycle 2	24	37.5	5.4 mg/mL min	No side effects
Cycle 3	24	37.5	5.7 mg/mL min	No side effects

During the next chemotherapy cycles, the patient was treated with the same cisplatin dose as the first cycle. The cisplatin dose was not escalated, because the patient had just recovered from the toxicity after the carboplatin dose of cycle 1. For cisplatin, an AUC_0–inf,free_ of 14.3 mg/L h was measured in chemotherapy cycle 3. Because of the high carboplatin AUC and observed clinical toxicity in cycle 1, the dose of carboplatin was reduced to 50% of the previous dose for cycles 2 and 3. After two carboplatin doses of 24 mg (37.5 mg/m^2^), AUC_0–inf,free_ values of 5.4 and 5.7 mg/mL min were measured in cycles 2 and 3, respectively. The second and third chemotherapy cycles were well tolerated.

Doxorubicin was administered in a dose of 30 mg/m^2^ on the day of carboplatin and the day after. During the first course, 100% of the recommended doxorubicin dose was administered, since the renal clearance of this drug is minimal. After development of toxicity in cycle 1, the dose of doxorubicin was reduced to 75% in cycles 2 and 3. Plasma concentrations of doxorubicin were not measured.

NIPD was started 3–4 h after end of infusion of cisplatin and 8–9 h after end of infusion of carboplatin. Platinum concentrations in the dialysate showed that platinum can be excreted by peritoneal dialysis. In total, approximately an amount of 0.4–0.5 mg cisplatin and 3–5 mg carboplatin was excreted after the first NIPD course after administration of cisplatin and carboplatin. This equals 3–4% of the total dose of cisplatin and 10–12% of the total dose of carboplatin.

### Treatment response and toxicity

The first response evaluation was performed using an MRI scan of the liver showing stable disease. Overall, the chemotherapy was well tolerated, but the patient developed grade 3 mucositis after the first cycle of carboplatin/doxorubicin. The AFP was reduced to 16,000 µg/L (after rise before treatment to 51,000 µg/L). Two additional courses of cisplatin and carboplatin/doxorubicin were given, his clinical condition improved during this period and NIPD was continued.

Thereafter, an uncomplicated left-sided extended hemihepatectomy was performed. NIPD was converted to continuous venovenous hemodiafiltration for 4 weeks postoperatively, after which NIPD was resumed.

Histology showed multifocal pure epithelial hepatoblastoma with fetal (well differentiated, crowded and pleomorphic) and embryonal differentiation. There was extensive necrosis with also vital tumor tissue (40% approx.) and macroscopic angio-invasion. Radical resection was obtained. Currently, he has finished his hepatoblastoma treatment and is in complete remission. Six months after the end of treatment, no signs of platinum-related neurotoxicity or ototoxicity has been observed.

## Discussion

This case report describes the pharmacokinetics of cis- and carboplatin in a child requiring peritoneal dialysis. These results show that it is feasible to measure AUCs for both drugs and to individualize the dose of these drugs according to the clinical parameters and PK results, even though consensus for the target AUC of cisplatin is lacking. In this case, AUCs of cisplatin of 15.3 and 14.3 mg/L h were well tolerated. Previously published, well tolerated, AUCs vary from 5.3 to 79.3 mg/L h in infants with hepatoblastoma (both with and without hemodialysis) [11, 12]. In this case, we chose not to escalate the dose, since the patient had just recovered from severe toxicity following the first carboplatin cycle.

Sebestyen et al. [4] reported a case of cisplatin treatment in a 2-year old patient receiving peritoneal dialysis. For this patient, an AUC of 64.1 and 66.6 mg/L h was measured after a dose of 25 mg/m^2^ and 29.7 mg/L h after a dose of 8.7 mg/m^2^. According to their data, peritoneal dialysis contributed less than 1% to total body clearance of cisplatin. They compared the values to the data of Dominici et al. [13], who reported an AUC of 15.5 ± 9.1 mg/L h in children with normal kidney function. The results of the AUC in our patient closely match the AUC of Dominici et al. However, the AUC values from the study of Dominici et al. were normalized to a dose of 100 mg/m^2^, while our patient was supposed to receive a cisplatin dose equal to 80 mg/m^2^.

Carboplatin was dosed according to the Newell formula [6]. Newell et al. developed a formula suited for GFR-based carboplatin dosing in children. The carboplatin dose is calculated using the target AUC, estimated GFR and body weight. For the first cycle, an estimated creatinine clearance of 5 ml/min was included in the Newell formula. A dose of 48 mg was given. This resulted in an AUC of 9.8 mg/mL min, 1.5-fold higher than the target AUC of 6.5 mg/mL min. At the same time, toxicity (grade 3 mucositis) was observed, so the dose was reduced to 50% of the previous dose. When dialytic clearance was actually measured, it was shown to be 3.6 ml/min (36 L/week).

A previous case report by English et al. [2] also used the Newell formula to calculate the carboplatin dose for a 4.3-year-old girl, diagnosed with Wilms tumor, receiving peritoneal dialysis. This patient had a residual renal clearance of 5 ml/min/1.73 m^2^ as determined by ^51^Cr EDTA clearance. According to their data, peritoneal dialysis did not contribute to carboplatin clearance. In our case, assuming no dialytic clearance of carboplatin would have led to a starting dose of 33 mg. In retrospect, this would have been more appropriate in this case; however, it can lead to under-dosing in patients who have residual renal function. Our advice for future cases would be to assume a dialytic clearance of zero to calculate the starting dose of carboplatin, and to adjust the dose if required, based on therapeutic drug monitoring.

The first carboplatin dose led to severe toxicity, which can be explained by the high AUC. Since carboplatin was administered during the same course as doxorubicin it is hard to distinguish whether the toxicity was caused by carboplatin or doxorubicin. The second and third courses, with reduced doses of carboplatin and doxorubicin, were well tolerated.

In addition, platinum concentrations in the dialysate were measured. These results show that 3–4% of the total dose of cisplatin and 10–12% of the total dose of carboplatin was excreted by peritoneal dialysis. This supports our hypothesis that free platinum can be excreted via peritoneal dialysis. The difference in amount between these compounds can be explained by the fact that cisplatin rapidly binds to proteins, faster than carboplatin. The half-lives of free cisplatin and carboplatin are approximately 0.5–1 h and 3–5 h, respectively. Furthermore, the time between administration of the platinum drugs and start of peritoneal dialysis is important. If peritoneal dialysis starts directly after end of infusion, it is expected that more free platinum can be excreted than when it starts several hours after end of infusion, since most of the drugs will be bound to proteins.

The clinical course of this 3-year-old patient with hepatoblastoma on NIPD has been astounding. These ‘tailored made’ and targeted chemotherapy courses enabled extended hemihepatectomy. He is currently off treatment and in complete remission.

In conclusion, this report shows that treatment with cis- and carboplatin in patients requiring peritoneal dialysis is possible and that pharmacokinetic monitoring contributes to the knowledge about the dosing of these drugs in patients requiring peritoneal dialysis.

## Data Availability

Not applicable.
